# Incidence Rates of Infections in Rheumatoid Arthritis Patients Treated with Janus Kinase or Interleukin-6 Inhibitors: Results of a Retrospective, Multicenter Cohort Study

**DOI:** 10.3390/jcm13103000

**Published:** 2024-05-20

**Authors:** Shuhei Yoshida, Masayuki Miyata, Eiji Suzuki, Takashi Kanno, Yuya Sumichika, Kenji Saito, Haruki Matsumoto, Jumpei Temmoku, Yuya Fujita, Naoki Matsuoka, Tomoyuki Asano, Shuzo Sato, Kiyoshi Migita

**Affiliations:** 1 Department of Rheumatology, Fukushima Medical University School of Medicine, 1 Hikarigaoka, Fukushima 960-1295, Japan; shuhei-y@fmu.ac.jp (S.Y.); ysumiti@fmu.ac.jp (Y.S.); s3xbck2p@fmu.ac.jp (K.S.); haruki91@fmu.ac.jp (H.M.); temmoku@fmu.ac.jp (J.T.); fujita31@fmu.ac.jp (Y.F.); naoki-11@fmu.ac.jp (N.M.); asanovic@fmu.ac.jp (T.A.); shuzo@fmu.ac.jp (S.S.); 2 Department of Rheumatology, Fukushima Red Cross Hospital, Yashima 7-7, Fukushima 960-8136, Japan; fukuintyoumm@fukushima-med-jrc.jp; 3 Department of Rheumatology, Ohta Nishinouchi General Hospital Foundation, 2-5-20 Nishinouchi, Koriyama 963-8558, Japan; azsuzuki@ohta-hp.or.jp (E.S.); t-kanno@ohta-hp.or.jp (T.K.)

**Keywords:** rheumatoid arthritis, Janus kinase inhibitor, interleukin-6 inhibitor, infection, tofacitinib, baricitinib, interstitial lung disease

## Abstract

**Objective:** This study aimed to compare the incidence rates (IRs) of infections, including herpes zoster (HZ), in rheumatoid arthritis (RA) patients treated with Janus kinase inhibitors (JAKis) or interleukin-6 inhibitors (IL-6is). **Methods:** We retrospectively analyzed 444 RA patients treated using IL-6is (*n* = 283) or JAKis (*n* = 161). After adjusting for clinical characteristic imbalances by propensity score matching (PSM), we compared the IRs of infections including HZ between the JAKi and IL-6i groups. **Results:** Observational period: 1423.93 patient years (PY); median observational period: 2.51 years. After PSM, incidence rate ratios comparing JAKi with IL-6i were 3.45 (95% confidence interval [CI]: 1.48–9.04) for serious infections other than HZ indicating that the JAKi-treated group was more likely to develop serious infection than the IL-6i-treated group. Multivariate Cox regression analyses revealed that the use of prednisolone > 5.0 mg/day, coexisting interstitial lung disease (ILD), and diabetes mellitus (DM) were independent risk factors for serious infections. The crude IR for HZ was significantly higher in the JAKi group, but the difference between groups was not significant (IRR: 2.83, 95% CI: 0.87–10.96) in PSM analysis. Unadjusted and PSM analyses performed in our study showed increased IRs of serious infections in patients with RA treated with JAKis compared with those treated with IL-6is. **Conclusions:** The presence of ILD or DM and the use of prednisolone were found to be independent risk factors for serious infection in RA patients treated using JAKis. Whereas the IRs for HZ after PSM were not significantly different between the JAKi and IL-6i groups.

## 1. Introduction

Targeting the Janus kinase (JAK) family with small-molecule inhibitors has proven effective in the treatment of various autoimmune diseases [1]. JAK inhibitors (JAKis) with different specificities for each JAK family have been approved for treating rheumatoid arthritis (RA) [2]. In the recent European Alliance of Associations for Rheumatology (EULAR) recommendations for RA management, JAKis are advised in patients who fail to respond to initial treatment with methotrexate (MTX) or other conventional synthetic disease-modifying antirheumatic drugs (csDMARDs) and have poor prognostic factors [3]. However, the safety concerns associated with JAKis, including infections and malignancies, should be carefully addressed. The risk of contracting serious infections or infections that require hospitalization is 1.5 to 2 times higher in patients with RA compared to the general population [4,5]. Similar to biological DMARDs (bDMARDs), JAKis can lead to serious and opportunistic infections including viral infections. Previous studies of JAKi have revealed elderly age, glucocorticoid usage (prednisolone ≥ 7.5 mg/day), diabetes mellitus, and high JAKi dosages as risk factors for infection [6]. However, the relative risk of infection in patients receiving JAKis was reported to be comparable to that in patients taking bDMARDs [7]. One retrospective cohort study revealed no significantly increased risk of serious infectious diseases related to the use of tofacitinib, a JAKi, compared with that associated with the use of a tumor necrosis factor inhibitor (TNFi) [8]. The infection profiles of TNFis and JAKis have been found to be similar with regard to serious infections [9,10]. Compared with bDMARDs, JAKis were found to be more frequently associated with the development of HZ [6,11,12]. In patients with RA who received tofacitinib in two phase I, nine phase II, six phase III, and two long-term extension (LTE) studies, the crude incidence rate (IR) of HZ was found to be 4.0 (95% confidence interval [CI]: 3.7, 4.4) per 100 patient years (PY) [13].

Given the widespread use of JAKis in the treatment of RA, the risk of infections, including HZ, in real-world settings should be addressed. Furthermore, it would be of great interest to compare the risks of infection between patients with RA receiving non-TNFi bDMARDs and those receiving JAKis in real-world clinical practice. Therefore, we conducted this multicenter cohort study to determine and compare the IRs of infection in patients with RA treated with an interleukin-6 inhibitor (IL-6i) or a JAKi in real-world settings.

## 2. Materials and Methods

### 2.1. Patients and Study Design

We have previously conducted a cohort study involving patients with RA attending our institution from April 2012 to December 2022 to compare the incidence of malignancy and major adverse cardiovascular events [14]. In the present study, we extended the inclusion period by 8 months until August 2023 and collected data on the incidence of serious infections and HZ. The cohort comprised patients treated at the Department of Rheumatology of Fukushima Medical University Hospital, Japanese Red Cross Fukushima Hospital, and Ohta Nishinouchi General Hospital Foundation. The study was approved by the institutional review boards of these institutions (approval Nos. 2021-157, 55, and 2022–8, respectively).

**Figure 1:**
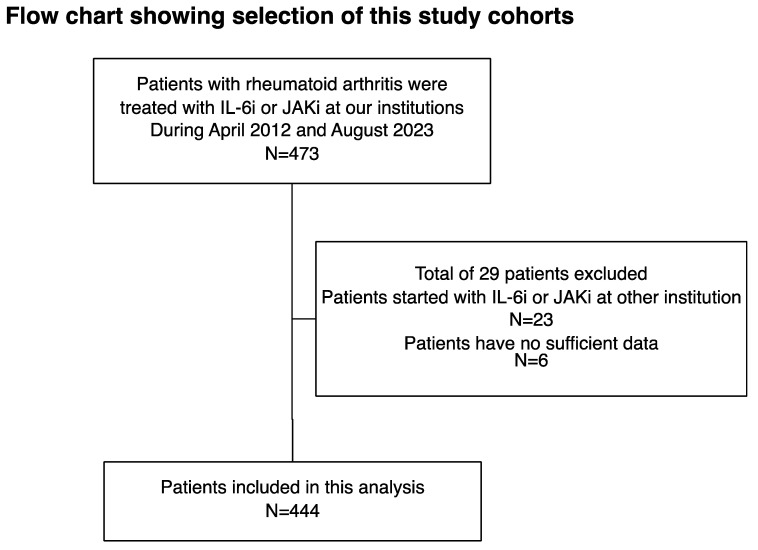
Flow chart showing patient enrollment. Among the 473 patients with RA who received initial treatment with IL-6i or JAKi at our institution between April 2012 and August 2023, 444 patients with sufficient clinical data were enrolled in the study. IL-6i: interleukin-6 inhibitor; JAKi: Janus kinase inhibitor; RA: rheumatoid arthritis.

Between April 2012 and August 2023, IL-6i or JAKi therapy was initiated in 473 patients with RA. Among them, 450 started receiving IL-6i or JAKi therapy at our institution, and 444 with sufficient clinical data were enrolled. All patients were diagnosed with RA according to the 2010 American College of Rheumatology/European League Against Rheumatism classification criteria for RA [15]. At treatment initiation, the collected demographic information included age, sex, disease duration, rheumatoid factor, anti-citrullinated protein antibodies, prior use of bDMARDs, presence of comorbidities such as DM or lung disease, and concurrent medications. Patients in the IL-6i group were administered tocilizumab either through intravenous infusion at a dosage of 8 mg/kg every 4 weeks or subcutaneous injection at 162 mg every other week, or sarilumab via subcutaneous injection at 200 mg every 2 weeks. Patients in the JAKi-treated group received the following dosages based on their specific conditions: those with renal impairment were given baricitinib 2 or 4 mg once daily, those with liver impairment received tofacitinib 5 mg twice or once daily, those with renal impairment were administered upadacitinib 15 mg once daily, and filgotinib was given at 100 or 200 mg once daily. The study employed an opt-out approach, and participants who refused to provide informed consent were not included. The full recruitment process is depicted in Figure 1.

### 2.2. Definitions of Exposure and Outcomes

“Exposure” was defined as the period from the initiation of IL-6i or JAKi treatment until treatment discontinuation, patient transfer to another hospital, death, or the end of the study period, whichever occurred first. Serious infections were defined as infections other than HZ that required hospitalization, as determined by treating physicians based on a comprehensive evaluation, including physical, laboratory, and radiological examinations, and the need for hospitalization. HZ was diagnosed by the treating physician based on the observation of skin lesions. The censoring time of the above-described adverse events was defined as the time from the administration of the first dose of JAKi or IL-6i until the end of treatment or the last observation point (31 August 2023).

### 2.3. Statistical Analysis

Data are presented as median and interquartile range for continuous variables and frequency and percentage for qualitative variables. The Mann–Whitney U test was employed to compare continuous variables, and Fisher’s exact test was used to compare categorical variables. Statistical significance was determined by a two-tailed *p*-value of <0.05.

To calculate the propensity scores, multivariable logistic regression analysis was performed with JAKi use as the dependent variable and the following as independent variables: patient age and sex, disease duration, RF and ACPA positivity, GC and MTX use, and co-occurrence of ILD and DM. Patient backgrounds of the treatment groups were adjusted via propensity score matching. The number of adverse events, PY at risk, and incidence rate ratio (IRR) with 95% CI were determined for each outcome.

The time to serious infection in the treatment groups was calculated using Kaplan–Meier analysis, and log-rank tests were employed to compare the cumulative IRs between the groups. Univariate and multivariate Cox regression analyses were conducted to identify variables related to the occurrence of serious infections. Factors with a *p*-value < 0.05 were included in the multivariate Cox regression analysis. The receiver operating characteristic (ROC) curve was used to determine the cut-off value for the steroid dose that impacted the risk of serious infection.

Statistical analyses were performed using R (version 4.1.2; R Foundation for Statistical Computing, Vienna, Austria, http://www.R-project.org/ [accessed on 23 June 2023]) and IBM SPSS Statistics software (version 29.0.1.0; IBM Co., Armonk, NY, USA).

## 3. Results

### 3.1. Patients’ Baseline Characteristics

Among the 473 patients with RA in whom IL-6i or JAKi treatment was initiated at our institutions between April 2012 and August 2023, 444 were enrolled. Table 1 presents the background characteristics of patients in the IL-6i- and JAKi-treated groups before and after propensity score matching (PSM). In the IL-6i-treated group, 277 and 6 patients received tocilizumab and sarilumab, respectively. The JAKi-treated group included 95, 43, 15, and 8 patients who received baricitinib, tofacitinib, upadacitinib, and filgotinib, respectively. None of the patients had a history of using JAKi. Before PSM, the IL-6i-treated group showed significantly higher concomitant GC use, GC and MTX doses, and longer observation periods compared to the JAKi-treated group. In contrast, the JAKi-treated group had significantly higher age at tsDMARD introduction and rates of DM coexistence. The observational period for the 444 patients (306 females) examined herein was 1423.93 PY, with a median (interquartile range) duration of 2.51 (1.20–4.22) years. After PSM, 236 patients with RA (165 females) were observed for 650.49 PY, with a median (interquartile range) length of 2.24 (1.22–3.97) years. Following PSM, no significant intergroup differences were observed, except for the history of bDMARD use and observational period.

### 3.2. IRs of Serious Infections

**Figure 2:**
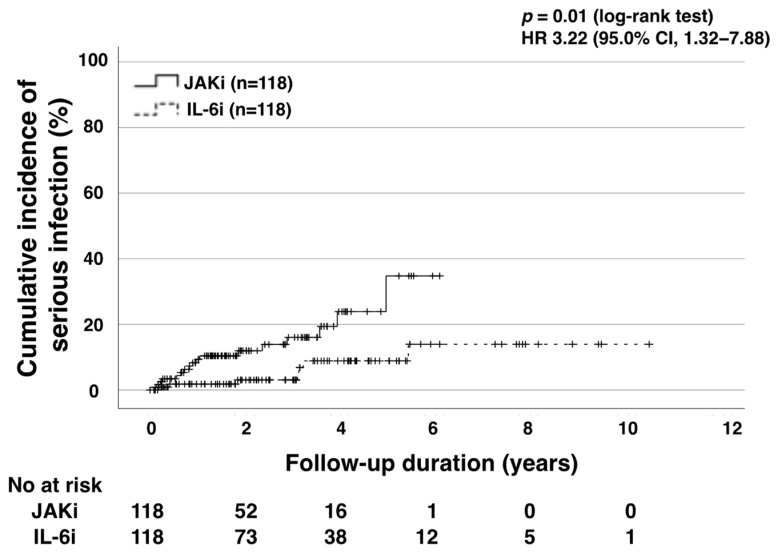
Cumulative incidence curves of serious infection in IL-6i-treated and JAKi-treated patients after propensity score matching. Kaplan–Meier curves show the cumulative incidence of serious infection in patients treated with IL-6is (*n* = 118) and JAKis (*n* = 118). Significant differences were observed between IL-6i-treated and JAKi-treated groups (*p* = 0.01). The starting point (0 years) was the date on which the observations began. HR: hazard ratio; CI: confidence interval; JAKi: Janus kinase inhibitor; IL-6i: interleukin-6 inhibitor; No: number.

The IRs for infectious diseases are listed in Table 2. We identified 27 and 25 cases of serious infections (16.8%; IR: 7.52/100 PY; 8.8%; IR: 2.35/100 PY) among 161 and 283 JAKi- and IL-6i-treated patients, respectively. Serious infections in the IL-6i group included bacterial pneumonia (*n* = 12), soft tissue infection (*n* = 6), pyelonephritis (*n* = 2), diverticulitis (*n* = 2), sinus mycosis (*n* = 1), pyothorax (*n* = 1), and bacterial enteritis (*n* = 1). In the JAKi group, these included bacterial pneumonia (*n* = 15), soft tissue infections (*n* = 5), bacterial enteritis (*n* = 2), pyelonephritis (*n* = 2), diverticulitis (*n* = 1), perforated peritonitis (*n* = 1), and cholecystitis (*n* = 1). Pneumonia (*n* = 27; 51.9% of all infections) was the most frequent serious infection in both groups. Before PSM, the IRR for the JAKi to the IL-6i group was 3.20 (95% CI: 1.85–5.56, *p* < 0.001), indicating that the former was more likely to develop serious infections. After PSM, the JAKi group was still more likely to develop serious infections (IRR: 3.45, 95% CI: 1.48–9.04, *p* = 0.004). However, the observational period for the JAKi group was shorter than that for the IL-6i group. Owing to this, we evaluated the time-to-event outcomes (serious infections) using Kaplan–Meier analysis. To this end, serious infections were more frequent in the JAKi group (*p* = 0.01, log-rank test; Figure 2).

### 3.3. Risk Factors for Serious Infections in JAKi-Treated Patients

**Figure 3:**
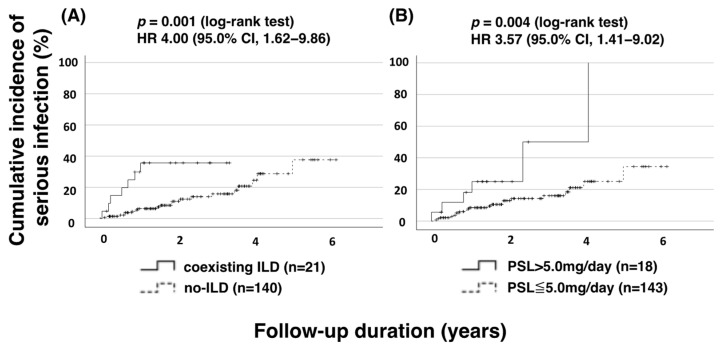
Cumulative incidence curves of serious infections in JAKi-treated patients with or without ILD and with PSL above and below 5.0 mg/day. Kaplan–Meier curves show the cumulative incidence of serious infections in RA patients treated with JAKis, stratified by (**A**) ILD status and (**B**) PSL > 5.0 mg/day and ≤5.0 mg/day. Significant differences were observed between the groups with and without ILD (*p* = 0.001). There was also a significant difference between the two groups in the dosage of PSL (PSL > 5.0 mg and PSL ≤ 5.0 mg) (*p* = 0.004). The starting point (0 years) was the date on which the observations began. HR: hazard ratio; CI: confidence interval; ILD: interstitial lung disease; PSL: prednisolone; JAKi: Janus kinase inhibitor.

To establish risk factors associated with serious infections, patient baseline characteristics were analyzed using univariate and multivariate Cox regression analyses (Table 3). Univariate Cox regression analysis revealed a significantly increased risk of serious infections associated with glucocorticoid (GC) use, interstitial lung disease (ILD), and diabetes mellitus (DM). In the multivariate Cox hazard model, prednisolone dose, ILD, and DM emerged as independent risk factors for serious infections in JAKi-treated patients. We analyzed the cut-off value for the GC dose associated with risk of serious infection by obtaining the ROC curve, which revealed a dose of 5.0 mg (Supplementary Figure S1). Therefore, Kaplan–Meier survival curves were plotted for occurrence of the first serious infection stratified by GC use (>5.0 mg/day) or the existence of ILD (Figure 3). The frequency of occurrence of the first serious infection was significantly higher in patients with ILD or those receiving high-dose GCs (>5.0 mg/day).

### 3.4. IR for HZ

The IRs for infectious diseases are listed in Table 2. The crude IRs for HZ were higher in the JAKi group (6.2%; IR: 2.91/100 PY) than in the IL-6i group (4.2%; IR: 1.13/100 PY). The crude IRs of HZ were significantly higher in the JAKi group compared to the IL-6i group (IRR = 2.49, 95% CI: 1.04–5.83, *p* = 0.041). After PSM, there was no significant difference in the IRR of HZ between the two groups (IRR = 2.83, 95% CI: 0.87–10.96, *p* = 0.084).

### 3.5. Comparison of Infectious Diseases between Each JAKi

The baseline demographic and clinical features of patients treated with each JAKi are summarized in Table 4. All “Serious infections other than HZ” and “HZ” in the table are those that occurred during JAKi administration. Among the JAKi group, serious infections occurred only in patients treated with baricitinib and tofacitinib. The baricitinib and tofacitinib groups are presented in Table 5. The crude IRs for serious infections or HZ were higher in the tofacitinib group than in the baricitinib group. However, there was no significant difference in the IRs for serious infection and HZ between these two groups (serious infection IRR = 1.33, 95% CI: 0.59–2.85, *p* = 0.48/HZ IRR = 1.92, 95% CI: 0.43–8.51, *p* = 0.38).

## 4. Discussion

In this study, we focused on patients with RA treated using targeted DMARDs and compared the incidences of infectious events, including HZ, between patients treated with JAKis and those treated with IL-6is. Despite the different baseline characteristics, our data demonstrated that JAKi-treated patients had a higher risk of serious infections than those treated with IL-6is. Although we could not completely exclude residual or unmeasured confounding factors, we also found increased IRs of serious infectious diseases in JAKi-treated patients with RA compared with those in IL-6i-treated patients with RA in our propensity score-matching comparisons.

The pooled data of baricitinib clinical trials showed that the IR for serious infectious diseases was 2.9/100 PY (95% CI: 2.5–3.4) [16]. In tofacitinib-treated patients with RA in phase I, phase II, phase III, and LTE studies, the IR for serious infections was reported to be 2.7/100 PY (95% CI: 2.5, 3.0), and the most common infection was pneumonia [17]. Elderly age, diabetes mellitus, corticosteroid use (>7.5 mg/day of prednisolone), and tofacitinib dosage (10 mg bid vs. 5 mg bid) were identified as risk factors for serious infections in patients treated with tofacitinib [6]. In contrast to these previous studies, in our study, the IR for serious infection (7.52/100 PY) was higher in patients treated with JAKis. Patients with RA at risk for infections, including elderly patients and those with diabetes or ILD, were enrolled in our real-world study, which could have contributed to the increased IRs for serious infections in JAKi-treated patients compared with those in clinical trial data. In general, GC use is associated with increased susceptibility to infection [18]. However, there are inadequate data on the effects of ILD on infection. We also evaluated the risk factors for serious infections in patients with RA receiving these targeted DMARDs. Our study clearly demonstrates that ILD is an independent risk factor for serious infectious diseases in JAKi-treated RA patients.

Controversy exists as to whether the use of JAKis for patients with RA-ILD can be challenging. Cronin et al. reported that the use of JAKis for the treatment of patients with RA and existing ILD did not increase the rate of hospitalization due to respiratory causes compared with rituximab treatment, suggesting that JAKi is a safe treatment strategy for RA patients with ILD [19]. However, the incidence rates of serious infectious diseases including pneumonia were significantly higher in JAKi-treated patients than in those treated with IL-6i in our study. Furthermore, the presence of ILD has been shown to be an independent risk factor for serious infectious diseases in JAKi-treated patients. Kalyoncu et al. found that in a controlled real-world study on tofacitinib-treated RA patients, infection was the most common cause of drug discontinuation in RA-ILD patients (more so than in non-RA-ILD patients) [20]. They also reported that RA patients with ILD are older than those without ILD. In general, elderly RA patients have a higher rate of comorbid ILDs than younger patients [21]. The possibility that older age and ILD comorbidity are confounding factors cannot be ruled out. In addition to old age, coexisting lung diseases and GC use were associated with the development of pneumocystis pneumonia in JAKi-treated patients [22]. Collectively, these data suggest that clinicians should be concerned about the risk of serious infections, including unusual infections, in patients receiving JAKis. Larger prospective studies are required to determine whether JAKis affect the risk of serious infections in RA patients with or without ILD.

A previous study on younger RA patients (<65 years) initiated on b/tsDMARDs, revealed that patients with frailty were at a significantly higher risk of serious infections than those without frailty [23]. Diabetes and interstitial lung disease are closely associated with patient frailty [24,25]. Therefore, RA patients with DM or ILD, even at a young age, should be cautious with JAKi treatment due to the close association between risk of developing serious infections and JAKi treatment.

Patients with RA have an approximately two- to three-fold increased risk of HZ compared with the general population [26]. In a systematic review and meta-analysis of JAKi-treated patients with RA, Bechman et al. demonstrated that the incidence of HZ was higher than that in the pooled placebo group (3.23/100 PY vs. 1.05/100 PY) [27]. Although the pathogenic mechanism by which JAKis increase the risk of HZ has not yet been completely elucidated, it is hypothesized that the inhibitory effect of JAKis on the intracellular signaling of cytokines acting via the JAK/signal transducer and activator of transcription (STAT) signaling pathway may contribute to the increased risk for HZ through the impairment of cell-mediated immunity [28]. In our study, the estimated IRs of HZ in patients receiving JAKi was 2.91/100 PY, (95% CI: 1.13–4.69) and was higher in the patients treated with JAKis than in those treated with IL-6is. This finding is consistent with those of previous studies, whereas the intergroup difference in our study was not significant. Given the lack of direct comparisons between JAKi-treated patients with RA and IL-6i-treated patients with RA with the same demographic background, we are limited in drawing conclusions regarding the relative risk of HZ linked to JAKis compared with that linked to IL-6is. However, a time-to-event analysis for HZ by using Kaplan–Meier curves showed that HZ developed more frequently in JAKi-treated patients compared with that in IL-6i-treated patients (Supplementary Figure S2). Therefore, physicians should be aware of the risk of HZ in JAKi-treated patients with RA, particularly those with risk factors.

Our study has several limitations. First, the number of patients (*n* = 473) and the duration of follow-up periods (median; 1.8 years in JAKi and 3.0 years in IL-6i) were not sufficient to detect any adverse events. Second, the choice of treatment and decision to discontinue treatment was made at the discretion of each rheumatologist, with no standardized protocol. Third, adverse events, including infections, were investigated at each patient visit, and the possibility that some cases were treated in the community and events were missed cannot be completely ruled out. Fourth, RA disease activity, HZ history, rate of vaccination, and parameters of a smoking history were not investigated in our study. Although high disease activity in RA has been reported to increase the risk of infection [29], it has been difficult to obtain information on disease activity from medical records. Vaccination for diseases such as HZ, influenza, and pneumococcal vaccines may interact with the IR of infection. Finally, the follow-up period was shorter for the JAKi group than for the IL-6i group.

## 5. Conclusions

In conclusion, our study demonstrated increased IRs for serious infectious diseases in JAKi-treated patients with RA compared with those in IL-6i-treated patients with RA in unadjusted and propensity score-matching analysis. Furthermore, we found that the presence of ILD or DM and the use of GCs (>5.0 mg/day) might be predictors of serious infections in JAKi-treated patients with RA. However, the IRs for HZ after PSM were not significantly different between the JAKi and IL-6i groups. More safety studies with long-term follow-ups in real-world settings are needed to fully elucidate the safety profile of JAKi for the management of patients with RA in clinical practice.

## Figures and Tables

**Table 1:** Comparisons of clinical features between IL-6i group and JAKi group.

Characteristics	All Patients			Propensity-Matched Patients			
	IL-6i (*n* = 283)	JAKi (*n* = 161)	*p* Value	IL-6i (*n* = 118)	JAKi (*n* = 118)	*p* Value	SMD
Male, *n* (%)	89 (31.4)	49 (30.4)	0.92	34 (28.8)	37 (31.4)	0.78	0.06
Age at b/ts DMARDs introduction, † years	61 (51–69)	72 (65–81)	<0.001 *	68 (58–76)	69 (60–75)	0.59	0.07
Disease duration, † years	8.7 (3.5–15.4)	7.6 (3.5–15.9)	0.51	9.0 (3.3–15.7)	9.2 (4.2–16.8)	0.39	0.17
Steinbrocker’s stage, I/II/III/IV	105/79/37/45 no data, 17	62/41/16/34 no data, 8		35/40/13/23 no data, 7	48/27/10/29 no data, 4		
Steinbrocker’s class, I/II/III/IV	30/168/62/8 no data, 15	17/93/45/4 no data, 2		14/70/25/2 no data, 7	7/73/34/2 no data, 2		
RF positivity, *n* (%)	207 (73.1)	109 (67.7)	0.23	85 (72.0)	84 (71.2)	1.00	0.02
ACPA positivity, *n* (%)	212 (74.9)	112 (69.6) no data, 2	0.32	87 (73.7)	89 (75.4)	0.88	0.04
Concomitant GC use, *n* (%)	134 (47.3)	41 (25.5)	<0.001 *	38 (32.2)	37 (31.4)	1.00	0.02
Concomitant GC dose, † mg/day	0.0 (0–5.0)	0.0 (0–0)	<0.001 *	0.0 (0.0–5.0)	0.0 (0.0–6.0)	0.55	0.09
Concomitant MTX use, *n* (%)	148 (52.3)	73 (45.3)	0.17	57 (48.3)	58 (49.2)	1.00	0.02
Concomitant MTX dose, † mg/week	4.0 (0.0–8.0)	0 (0–6)	0.04 *	0.0 (0.0–6.0)	0.0 (0.0–6.0)	0.91	0.03
Coexisting ILD, *n* (%)	37 (13.1)	21 (13.0)	1.0	14 (11.9)	15 (12.7)	1.00	0.03
Coexisting DM, *n* (%)	26 (9.2)	26 (16.1)	0.03 *	17 (14.4)	16 (13.6)	1.00	0.02
Previous use of bDMARDs, *n* (%)	113 (39.9)	76 (47.2)	0.16	44 (37.3)	61 (51.7)	0.04 *	0.29
Observational period, † years	3.0 (1.4–5.2)	1.8 (1.2–3.3)	<0.001 *	2.9 (1.4–4.7)	1.9 (1.2–3.3)	0.01 *	0.45

† Values are the median with interquartile range. * means there is a significant difference at *p* < 0.05. IL-6i: interleukin-6 receptor inhibitors, JAKi: Janus kinase inhibitors, RF: rheumatoid factor, ACPA: anti-citrullinated peptide antibody, GC: glucocorticoid, MTX: methotrexate, ILD: interstitial lung disease, DM: diabetes mellitus, b/ts DMARD: biologic/targeted synthetic disease-modifying anti-rheumatic drug, SMD: standardized mean difference.

**Table 2:** Incidence rate of infectious diseases.

	All Patients			Propensity-Matched Patients		
	IL-6i (*n* = 283)	JAKi (*n* = 161)	*p* Value	IL-6i (*n* = 118)	JAKi (*n* = 118)	*p* Value
Serious infectious diseases other than HZ	25 (8.8)	27 (16.8)		7 (5.9)	17 (14.4)	
IR per 100 PY (95%CI)	2.35 (1.42–3.28)	7.52 (4.73–10.31)		1.89 (0.50–3.28)	6.61 (3.57–9.65)	
IRR (95%CI)	1 [reference]	3.20 (1.85–5.56)	<0.001 *	1 [reference]	3.45 (1.48–9.04)	0.004 *
HZ	12 (4.2)	10 (6.2)		4 (3.4)	8 (6.8)	
IR per 100 PY (95%CI)	1.13 (0.48–1.78)	2.91 (1.13–4.69)		1.08 (0.03–2.13)	3.11 (0.99–5.23)	
IRR (95%CI)	1 [reference]	2.49 (1.04–5.83)	0.041 *	1 [reference]	2.83 (0.87–10.96)	0.084

* means there is a significant difference at *p* < 0.05. IL-6i: interleukin-6 inhibitor; JAKi: Janus kinase inhibitor; HZ: herpes zoster, IR: incidence rate, PY: patient years, IRR: incidence rate ratio, CI: confidence interval.

**Table 3:** Independent risk factors of serious infectious diseases in rheumatoid arthritis patients treated with JAKi.

Risk Factors for Serious Infectious Diseases				
Variavle	Univariate Model		Multivariable Model	
	HR (95%CI)	*p*-Value	HR (95%CI)	*p*-Value
Age, >65 years or not	2.00 (0.69–5.81)	0.20		
Disease duration, per 1-year increase	0.98 (0.94–1.03)	0.42		
RF positive or negative	1.50 (0.63–3.56)	0.36		
ACPA positive or negative	1.88 (0.71–4.98)	0.21		
GC dose, per 1 mg increase	1.012 (1.01–1.02)	<0.001 *	1.01 (1.01–1.02)	<0.001 *
MTX dose, per 1 mg increase	0.956 (0.85–1.07)	0.45		
Coexisting ILD, yes/no	4.00 (1.62–9.86)	0.003 *	3.72 (1.48–9.35)	0.01 *
Coexisting DM, yes/no	2.68 (1.20–5.97)	0.02 *	2.53 (1.11–5.75)	0.03 *
No. of previous use of bDMARDs, per drug	1.10 (0.79–1.49)	0.63		
Reduced dose of JAKi, yes/no	0.74 (0.35–1.59)	0.44		

* means there is a significant difference at *p* < 0.05. JAKi: Janus kinase inhibitor, RF: rheumatoid factor, ACPA: anti-citrullinated peptide antibody, GC: glucocorticoid, MTX: methotrexate, ILD: interstitial lung disease, DM: diabetes mellitus, No: number, bDMARDs: biological disease-modifying antirheumatic drugs, HR: hazard ratio, CI: confidence interval.

**Table 4:** Clinical features of RA patients treated with each JAKi.

	Baricitinib (*n* = 95)	Tofacitinib (*n* = 43)	Upadacitinib (*n* = 15)	Filgotinib (*n* = 8)
Male, *n* (%)	25 (26.3)	12 (27.9)	8 (53.3)	4 (50.0)
Age at JAKi introduction, † years	74 (68–84)	72 (66–79)	61 (56–68)	64 (61–68)
Disease duration, † years	7.3 (3.1–16.5)	8.7 (4.1–15.6)	6.4 (3.8–8.6)	6.6 (4.2–12.2)
Steinbrocker’s stage, I/II/III/IV	36/21/12/20 no data 6	18/13/1/9 no data 2	5/6/2/2	3/1/1/3
Steinbrocker’s class, I/II/III/IV	15/53/23/3 no data 1	2/21/18/1 no data 1	0/11/4/0	0/8/0/0
RF positivity, *n* (%)	65 (68.4)	26 (60.5)	12 (80.0)	6 (75.0)
ACPA positivity, *n* (%)	68 (71.6) no data 1	26 (60.5) no data 1	12 (80.0)	6 (75.0)
Concomitant GC use, *n* (%)	18 (18.9)	11 (25.6)	9 (60.0)	3 (37.5)
Concomitant GC dose, † mg/day	0.0 (0.0–0.0)	0.0 (0.0–1.8)	2.0 (0.0–2.5)	0.0 (0.0–5.5)
Concomitant MTX use, *n* (%)	35 (36.8)	27 (62.8)	6 (40.0)	5 (62.5)
Concomitant MTX dose, † mg/week	0.0 (0.0–6.0)	4.0 (0.0–7.0)	0.0 (0.0–5.0)	5.0 (0.0–6.5)
Coexisting ILD, *n* (%)	13 (13.7)	6 (14.0)	2 (13.3)	0
Coexisting DM, *n* (%)	15 (15.8)	9 (20.9)	2 (13.3)	0
Previous use of bDMARDs, *n* (%)	44 (46.3)	23 (53.5)	8 (53.3)	1 (12.5)
Observation period, † years	1.8 (1.2–3.3)	2.1 (1.0–3.6)	1.9 (1.5–2.1)	1.3 (1.0–1.4)
Serious infectious diseases other than HZ, *n* (%)	16 (16.8)	11 (25.6)	0	0
HZ	4 (4.2)	4 (9.3)	2 (13.3)	0

† Values are the median with interquartile range. JAKi: Janus kinase inhibitors, RF: rheumatoid factor, ACPA: anti-citrullinated peptide antibody, GC: glucocorticoid, MTX: methotrexate, ILD: interstitial lung disease, DM: diabetes mellitus, bDMARD: biologic disease-modifying anti-rheumatic drug, HZ: herpes zoster.

**Table 5:** Incidence rates of serious infections, HZ.

	Baricitinib (*n* = 95)	Tofacitinib (*n* = 43)	*p*-Value
Serious infectious diseases other than HZ	16 (16.8%)	11 (25.6%)	
IR per 100 PY (95%CI)	7.54 (3.93–11.15)	9.95 (4.14–15.76)	
IRR (95%CI)	1 [reference]	1.33 (0.59–2.85)	0.48
HZ	4 (4.2%)	4 (9.3%)	
IR per 100 PY (95%CI)	1.89 (0.03–3.75)	3.62 (0.00–7.24)	
IRR (95%CI)	1 [reference]	1.92 (0.43–8.51)	0.38

HZ: herpes zoster, IR: incidence rate, PY: patient years, IRR: incidence rate ratio, CI: confidence interval.

## Data Availability

The raw data supporting the conclusions of this article will be made available by the authors, without undue reservation.

## Supplementary Materials

The following supporting information can be downloaded at: https://www.mdpi.com/article/10.3390/jcm13103000/s1, Supplementary Figure S1. The receiver operating characteristic curve representing the cut-off value of glucocorticoid dose in the prediction of serious infections. AUC: area under the curve. Supplementary Figure S2. Cumulative incidence curves of HZ in IL-6i-treated and JAKi-treated patients after propensity score matching. Kaplan–Meier curves show the cumulative incidence of HZ in patients treated with IL-6is (*n* = 118) and JAKis (*n* = 118). Significant differences were observed between IL-6i-treated and JAKi-treated groups (*p* = 0.024). The starting point (0 years) was the date on which the observations began. HZ: herpes zoster; JAKi: Janus kinase inhibitor; IL-6i: interleukin-6 inhibitor; CI: confidence interval; No: number.

## Abbreviations

ACPA	Anti-citrullinated protein antibodies
bDMARDs	Biologic disease-modifying antirheumatic drugs
CI	Confidence interval
csDMARDs	Bonventional synthetic disease-modifying antirheumatic drugs
DM	Diabetes mellitus
DMARDs	Disease-modifying antirheumatic drugs
EULAR	European Alliance of Associations for Rheumatology
GC	Glucocorticoid
HR	Hazard ratio
HZ	Herpes zoster
IL	Interleukin
IL-6i	Interleukin-6 inhibitor
ILD	Interstitial lung disease
IR	Incidence rate
IRR	Incidence rate ratio
JAK	Janus kinase
JAKi	Janus kinase inhibitor
MTX	Methotrexate
PSM	Propensity score matching
PY	Patient years
RA	Rheumatoid arthritis
RF	Rheumatoid factor
ROC	Receiver operating characteristic
STAT	Signal transducer and activator of transcription
TNFis	Tumor necrosis factor inhibitors
tsDMARDs	Targeted synthetic disease-modifying antirheumatic drugs
