# Supplementary figures and images for: Incidence Rates of Infections in Rheumatoid Arthritis Patients Treated with Janus Kinase or Interleukin-6 Inhibitors: Results of a Retrospective, Multicenter Cohort Study
Source: J Clin Med. 2024 May 20;13(10):3000. doi: 10.3390/jcm13103000 (PMC11122599; doi:10.3390/jcm13103000)

Supplementary Fig. 1

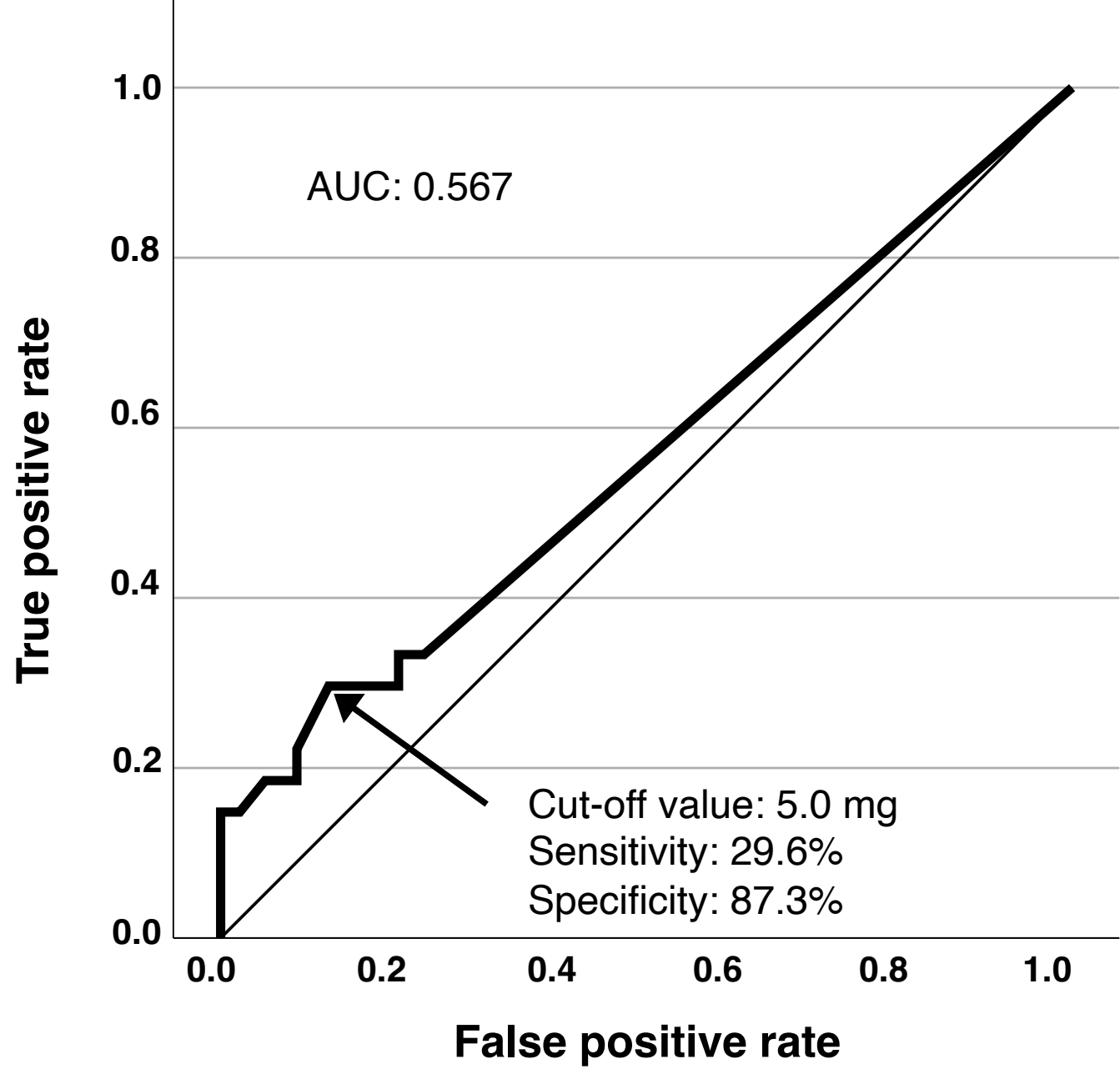

Supplement: Supplementary file 1 [file jcm-13-03000-s001.zip › Supplementary Fig. 1.pdf]

Supplementary Fig. 2

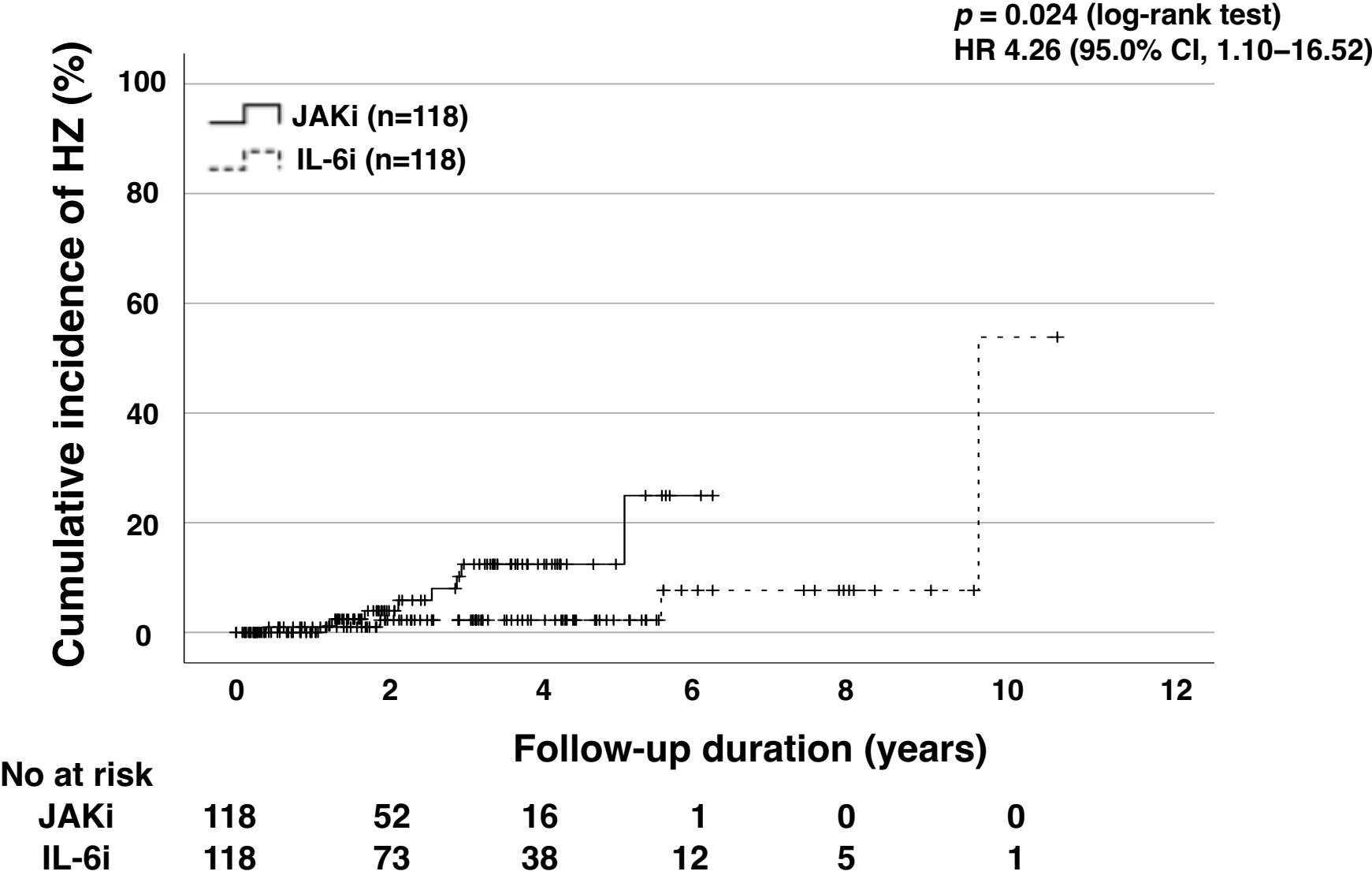

Supplement: Supplementary file 1 [file jcm-13-03000-s001.zip › Supplementary Fig. 2.pdf]
